# The Role of Ethnic-Specific Gender Schemas in Ethnic Disparities in Adolescent Girls’ Disruptive Behavior: A Preliminary Examination

**DOI:** 10.38207/jcmphr/2022/mar030205271a

**Published:** 2022-06

**Authors:** Colleen A. Halliday, Katherine A. Perkins, Claudia A. Salazar, Carla Kmett Danielson

**Affiliations:** 1 Division of Global and Community Health, Department of Psychiatry & Behavioral Science, Medical University of South Carolina; 2 Yvonne and Schuyler Moore Child Development Research Center, College of Education, University of South Carolina; 3 Group on Diversity Affairs, College of Medicine Dean’s Office, Medical University of South Carolina; 4 National Crime Victims and Treatment Center, Department of Psychiatry & Behavioral Science, Medical University of South Carolina

**Keywords:** African American, Girls, Ethnic/Racial Differences, Disruptive Behavior, Aggression, Gender Schemas

## Abstract

African American adolescent girls have evidenced higher levels of disruptive behavior than girls from other ethnic groups. However, most research focused on understanding disparities in these outcomes has been conducted without consideration of gender or has focused exclusively on boys. Yet, prior research suggests that anger and aggression are less gender-typed in African American youth than they are among youth from other ethnic backgrounds. The purpose of this preliminary investigation was to examine the extent to which ethnic-specific gender schemas about anger mediated the relationship between ethnicity and girls’ disruptive behavior. Participants were 66 middle school girls (24.1 % African American, 46.3 % European American; *M*_age_= 12.06). They completed measures of ethnic-specific gender schemas about anger, reactive and instrumental aggression, and classroom disruptive behavior. Results indicated that relative to girls from other ethnic groups, African American girls had higher levels of reactive aggression and classroom disruptive behavior, both of which are rooted in anger. In contrast, no ethnic difference was found for instrumental aggression, which is not connected to anger. Ethnic-specific gender schemas about anger at least partially accounted for ethnic differences in reactive aggression and classroom disruptive behavior. Findings highlight the importance of examining gender schemas specific to ethnicity as factors in ethnic disparities in behavioral outcomes among adolescent girls.

## Introduction

The disproportionate disruptive behavior outcomes in African American (AA) youth relative to the youth of other ethnic backgrounds have prompted research focused on understanding the factors contributing to ethnic disparities in youth aggression and other behavior problems. Most of this work has been conducted without consideration of gender or has focused exclusively on AA boys [1]. Yet, ethnic disparities may be even greater for girls than they are for boys. In a U.S. nationwide study, for example, AA boys were 1.67 times as likely to engage in physical fighting at school compared to European American (EA) boys, whereas AA girls were 4.4 times as likely as EA girls to engage in this behavior [10]. Consequently, research is needed that goes beyond addressing factors that explain ethnic disparities in disruptive behavior generally. An improved understanding of the unique factors contributing to ethnic disparities in disruptive behavior among girls is needed to inform treatment and prevention efforts to reduce disparities in this area.

Evidence suggests that aggression is less gender-typed in AA youth than it is among youth from other ethnic backgrounds [4]. According to gender schema theories [2,12], children develop schemas about their own gender that include beliefs about gender-appropriate behavior within their culture. Functionally, gender schemas are internalized gender stereotypes. Children view themselves, make decisions, and regulate their behaviors based on gender schemas. Because gender stereotypes vary over time and across ethnic groups, cultural context determines which behaviors are considered gender-typed. In dominant U.S. culture, displays of anger and physical aggression are generally stereotyped as the purview of boys and not girls [3]. However, AA adolescents have evidenced lower endorsement of conventionally held gender schemas of aggression than youth from other ethnic backgrounds [4]. Furthermore, AA girls and women are stereotyped as being hostile, argumentative, and aggressive [17]. As such, AA girls might be more likely than girls from other ethnic groups to have gender schemas that include anger and aggression as appropriate for girls. What remains unknown is the extent that ethnic-specific gender schemas about anger and aggression account for ethnic disparities in girls’ disruptive behavior.

The current study is a preliminary examination of the extent to which ethnic-specific gender schemas mediate the relationship between ethnicity and various forms of girls’ disruptive behavior. Middle school girls responded to questionnaires assessing reactive aggression, which is rooted in anger, and instrumental aggression, which is unrelated to anger [11]. They also completed self-report measures of disruptive classroom behavior and ethnic-specific gender schemas about anger. Established predictors of disruptive behavior, deviant peer affiliation, parental demandingness, and neighborhood income also were assessed to serve as covariates. We hypothesized that AA girls would evince more anger-related disruptive behavior than girls of other ethnic groups and that ethnic-specific gender schemas about anger would account for these differences. Specifically, we expected ethnic-specific gender schemas about anger to mediate the relationship between ethnicity and reactive aggression, but no such effects on instrumental aggression. Because anger at unfair treatment has been proposed as a factor underlying disruptive behavior at school more for AA girls than for girls of other ethnic groups [14], we also expected ethnic disparities in disruptive classroom behavior to be mediated by ethnic-specific gender schemas about anger.

## Methods

### Participants and Procedures

Participants were 66, 6^th^ to 8^th^ grade girls recruited from community settings in a Southeastern region in the U.S. Each participant completed questionnaires online, for which she received compensation. Ages ranged from 11 to 16 years (*M*_age_= 12.65, *SD*_age_ = 1.03). Ethnicities were Black/AA (*n* = 22, 33.3 %); non-Hispanic White/EA (*n* = 25, 37.9 %); multiethnic (*n* = 15, 22.7 %,); Hispanic/Latino (*n* = 2, 3.0 %); Asian/Asian American, (*n* = 1, 1.5 %); and other (*n* = 1, 1.5 %).

## Measures

### Ethnicity.

Participants reported their ethnicity. Responses were dichotomized to reflect AA ethnicity (1 = AA, 0 = non-AA).

### Aggression.

Aggression was assessed with Little and colleagues’ (2003) aggression questionnaire adapted to assess behavioral frequency. Two scores were computed: *reactive aggression* (α = .91; 12 items), which indexed angry aggressive defensive responses to peer transgressions, and *instrumental aggression* (α = .96; 12 items), which assessed aggressive behaviors enacted to achieve the desired outcome.

### Disruptive classroom behavior.

Disruptive classroom behavior was assessed using the Patterns for Adaptive Learning Scales [13] disruptive behavior subscale (5 items; α = .80).

### Ethnic-specific gender schemas about anger.

The Stereotypic Roles for Black Women [17] was developed and validated for adult women to assess the adoption of four stereotypes of Black women. Townsend and colleagues (2010) simplified this instrument for use with Black adolescent girls. Participants in the current study completed the anger subscale, adapted to be applicable to girls of any ethnic group by replacing the words “Black girls” with “girls of my ethnic group” (9 items; α = .87).

### Parent demandingness.

The demandingness subscale of the Parenting Styles Inventory-II [5] captured youth reports of parents’ setting and enforcing rules (5 items, α =.77).

### Neighborhood income.

The Federal Financial Institutions Examination Council’s (2017) income classification for the census tract of each participant’s residence indexed neighborhood income.

### Deviant peer affiliation.

Ettekal and Ladd’s (2015) school conduct problems subscale and [6] friend antisocial behavior scale were combined to measure peer deviance (9 items; α = .94).

## Results

In preliminary independent-samples *t*-tests, AA girls showed higher reactive aggression and disruptive classroom behavior compared to girls in other ethnic groups (**see** Table 1). Instrumental aggression did not significantly differ based on ethnic group. Subsequent mediation analyses, therefore, focused on reactive aggression and disruptive classroom behavior.

The PROCESS macro [9] for SPSS version 24 was used to test the hypothesis that ethnic-specific gender schemas about anger mediated the effects of ethnicity on both reactive aggression and classroom disruptive behavior. To account for the effects of established predictors of disruptive behavior, deviant peer affiliation, parental demandingness, and neighborhood income were included as parallel mediators. Indirect effects were estimated using the [15] bootstrapping method for each of 10,000 bootstrapped samples [9].

### Reactive Aggression

**Figures 1 (a, b):**
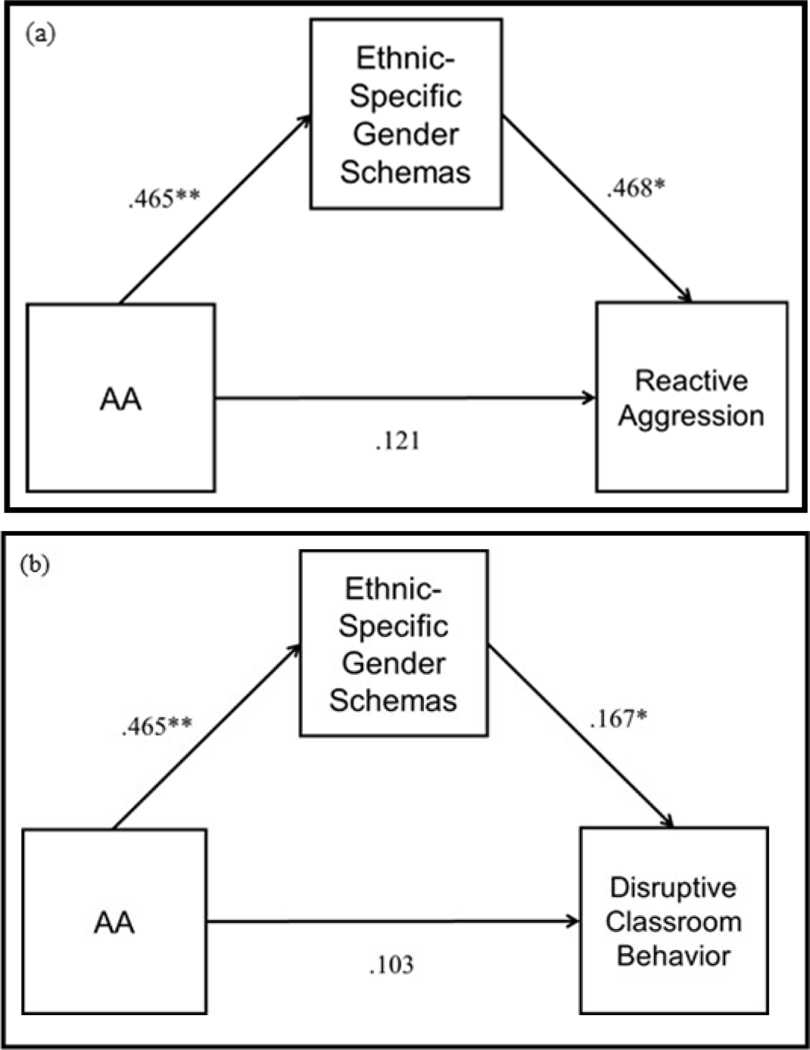
Path model depicting perceived ethnic-specific gender schemas as a mediator of the effect of ethnicity on (a) reactive aggression (b) disruptive classroom behavior, controlling for peer deviance, parent structure, and neighborhood income. Path values reflect standardized coefficients. * p < .05, ** p < .01

The overall model for predicting reactive aggression was significant, *F*(5, 59) = 10.92, *p* < .001, *R*^2^ = .693. The indirect effect of ethnicity on reactive aggression through ethnic-specific gender schemas about anger was significant at 95 % CI [.0420, .5168]. Thus, ethnic-specific gender schemas about anger mediated the relationship between ethnicity and reactive aggression. Results are presented in Table 2 and illustrated in Figure 1a.

### Disruptive Classroom Behavior

The overall model for predicting disruptive classroom behavior was significant, *F*(5, 59) = 4.87, *p* = .0009 *R*^2^ = .2921. The indirect effect of ethnicity on classroom disruptive behavior, through ethnic-specific gender schemas about anger was significant, 95 % CI [.0164, 0.1786]. Thus, ethnic-specific gender schemas about anger mediated the relationship between ethnicity and disruptive classroom behavior. Results are presented in Table 2 and illustrated in Figure 1b.

## Discussion

Consistent with current research [10] and hypotheses, AA girls reported higher levels of reactive aggression and disruptive classroom behavior than did girls of other ethnicities. Also as hypothesized, girls’ instrumental aggression did not differ based on ethnicity. Hence, ethnic disparities in the girls were found only for anger-related disruptive behavior. As hypothesized, AA girls had higher ethnic-specific gender schemas about anger than did other girls. This finding aligns with ethnic-specific gender stereotypes of anger and aggression. Prevailing Western stereotypes for girls do not include anger or aggression [3], whereas stereotypes for AA girls do [17].

As hypothesized, ethnic-specific gender schemas about anger mediated effects of ethnicity on reactive aggression and classroom disruptive behavior. These findings suggest that ethnic differences in gender schemas about anger for girls at least partially account for ethnic disparities in girls’ anger-related disruptive behavior. Unfortunately, members of ethnically stigmatized groups sometimes embrace and are influenced by negative stereotypes about their group [8]. If corroborated, findings suggest that some cognitions targeted in cognitive treatment of disruptive behavior are tied to identity characteristics (i.e., ethnicity and gender), making them particularly resistant to change [16]. Hence, tailoring interventions to address identity-related cognitions about anger may enhance treatment outcomes among AA girls.

## Limitations

A limitation of this preliminary investigation is its modest sample size. Consequently, non-AA girls were in a group primarily comprised of EA girls. Further, as a correlational, cross-sectional study, causation cannot not be determined. Moreover, all data came from adolescent self-reports. The study should be replicated with a larger, ethnically diverse sample, a longitudinal design, and multiple respondents, such as parents, teachers, and peers.

## Conclusion

Literature about gender schema for girls and women is based primarily on European American samples without consideration of ethnicity [3]. Because gender schemas are learned in cultural contexts, additional research is needed to the role that understanding ethnic-specific gender schemas play in disruptive behavior outcomes for African American girls. Research is also needed to improve the understanding of processes related to the internalization of negative ethnic stereotypes that are specific to one’s gender. More generally, this study highlights the need for future studies examining ethnic-specific gender factors in ethnic disparities in girls’ outcomes to inform efforts to reduce disparities in these outcomes.

## Figures and Tables

**Table 1:** Ethnic differences in variables

	African American (*n* = 22)		Non-African American (*n* = 44)		
Variable					*t*-test
	*M*	*SD*	*M*	*SD*	
**Reactive Aggression**	**3.23**	**1.26**	**1.90**	**1.65**	−3.32**
Instrumental Aggression	1.12	1.22	0.64	1.10	−1.62
**Disruptive Classroom Behavior**	**1.97**	**.715**	**1.45**	**.49**	−3.49**
**Deviant Peer Affiliation**	**1.67**	**1.06**	**.573**	**.62**	−4.48**
**Parent Demandingness**	**1.85**	**.549**	**1.53**	**.49**	−2.44*
**Ethnic-Specific Gender Schemas**	**3.05**	**.82**	**2.33**	**.72**	−3.48*
**Neighborhood Income**	**2.23**	**.81**	**3.14**	**.86**	4.12*

* *p* < .05

** p < .01

**Table 2:** Regression results for classroom disruptive behavior and reactive aggression

Predictor	Reactive Aggression		Classroom Disruptive Behavior	
	β	*SE*	β	*SE*
Ethnic-Specific Gender Schemas	**.467** **	**.178**	**.204** *	**.096**
Deviant Peer Affiliation	.317	.237	**.257** **	**.088**
Parent Demandingness	**.619** **	**.201**	−.137	.166
Neighborhood Income	.082	.183	.019	.085
African American	.121	.420	.204	.096

* *p* < .05

** p < .01
